# Effective treatment with daratumumab in post-HSCT refractory immune-mediated cytopenias: a case report and literature review

**DOI:** 10.3389/fimmu.2025.1625365

**Published:** 2025-08-01

**Authors:** Xiao-yu Jing, Dong-jun Li, Sheng-nan Su, Qing-kai Dai, Shu-wen Sun, Liang Huang, Yuan Ai, Ju Gao, Yi-Ping Zhu, Jia-qi Ni, Xiao-xi Lu

**Affiliations:** ^1^ Department of Pediatrics, West China Second University Hospital, Sichuan University, Chengdu, China; ^2^ Key Laboratory of Obstetric and Gynecologic and Pediatric Diseases and Birth Defects, Sichuan University, Ministry of Education, Chengdu, China; ^3^ West China School of Clinical Medicine, Sichuan University, Chengdu, China; ^4^ Department of Laboratory Medicine, West China Second University Hospital, Chengdu, China; ^5^ Department of Pharmacy/Evidence-Based Pharmacy Center, West China Second University Hospital, Sichuan University, Chengdu, China; ^6^ Key Laboratory of Birth Defects and Related Diseases of Women and Children, Sichuan University, Ministry of Education, Chengdu, China; ^7^ West China School of Pharmacy, Sichuan University, Chengdu, China; ^8^ NHC(National Health Commission) Key Laboratory of Chronobiology, Sichuan University, Chengdu, China

**Keywords:** immune-mediated cytopenias, post-HSCT, daratumumab, pediatric, immune-mediated thrombocytopenia

## Abstract

Immune-mediated cytopenias (IMCs) following allogeneic hematopoietic stem cell transplantation (HSCT) can lead to substantial morbidity and mortality, presenting a major therapeutic obstacle. Here, we report a case of a pediatric patient with acquired aplastic anemia. Nine months after HSCT, this patient developed severe, refractory hemolytic anemia and immune-mediated thrombocytopenia (IMT). Despite treatment with corticosteroids, intravenous immunoglobulin (IVIG), rituximab, along with avatrombopag, romiplostim, acetylcysteine, and decitabine, the patient’s platelet count showed no signs of improvement. Subsequently, daratumumab, a monoclonal antibody targeting CD38, was administered. This treatment induced a rapid and sustained response. Four months after initial daratumumab administration, the percentage of CD38-positive immune cells in the patient’s peripheral blood increased, which was concurrent with another decline in platelet levels. After re-initiating daratumumab therapy, the patient’s platelet count returned to normal levels. The only significant adverse effect noted was a delayed recovery of humoral immunity. Daratumumab, by targeting antibody-producing plasma cells, shows promise as a therapeutic alternative for refractory IMCs in post-HSCT patients.

## Introduction

The incidence of immune-mediated cytopenias (IMCs) following hematopoietic stem cell transplantation (HSCT) in patients with non-malignant conditions varies significantly across different studies. In pediatric cases, this incidence ranges from 1.5% to 22% as reported in several studies (1–3). These complications can manifest as immune-mediated hemolytic anemia (IMHA), immune-mediated thrombocytopenia (IMT), or immune-mediated neutropenia (IMN). They may occur independently or in combination, as seen in Evans syndrome, where immune hemolytic anemia coexists with thrombocytopenia or neutropenia. Among these, hemolytic anemia is the most common form of IMCs (4–6). Immune thrombocytopenia has been observed in 0.5-2% of pediatric HSCT patients (1, 7). Typically, IMCs develop within a median time frame of 2 to 40 months after HSCT, regardless of the continuous use of immunosuppressive therapy aimed at preventing or managing graft-versus-host disease (GVHD) (3, 8).

The pathophysiology of IMCs following HSCT remains incompletely understood. Risk factors for IMCs include conditioning regimen type, anti-T-cell serotherapy, recipient’s cytomegalovirus serostatus, umbilical cord blood stem cells, unrelated donor source, and GVHD development (9–11). The pathophysiology of IMCs involves humoral and cellular immune dysfunctions: autoreactive CD4+ (e.g., T follicular helper cells) and CD8+ T cells drive autoantibody production (e.g., anti-GPIIb/IIIa, GPIb/IX) and direct cytotoxicity against platelets/megakaryocytes. Autoantibodies trigger antibody-dependent cellular phagocytosis and complement-mediated cytotoxicity via the membrane attack complex, with the spleen (macrophages) and liver (desialylation via the Ashwell-Morell receptor) as key sites of platelet destruction. Regulatory T/B cell deficiencies and complement activation amplify immune responses. Genetic factors, such as human leukocyte antigen (HLA) and FcγR polymorphisms, and clonal hematopoiesis may influence susceptibility and treatment resistance, while impaired thrombopoietin signaling and megakaryocyte dysfunction reduce platelet production (12).

Although prednisolone and other immunosuppressive agents can control most cases of IMCs, up to 60% of patients with post-transplant IMT do not respond fully to first- or second-line therapies, including rituximab (13). The relapse mechanism is likely due to the re-emergence or continuous production of platelet auto-antibodies by pathogenic platelet-specific B cells, potentially long-lived plasma cells and plasma blasts that reside in the bone marrow, liver, and spleen. Plasma blasts and plasma cells strongly express CD38 rather than CD20, allowing them to evade CD20-directed therapy. Daratumumab, an anti-CD38 antibody designed for the treatment of multiple myeloma and approved by the FDA in 2015 (14), has the ability to target these antibody-producing plasma cells. It has shown promise in treating patients with refractory IMCs. Recently, there have been reports of successful use of daratumumab in managing resistant IMHA or IMT (15–19). Here, we present our experience of treating a pediatric patient with autoimmune cytopenias secondary to very severe aplastic anemia (VSAA) following allogeneic HSCT using daratumumab at our pediatric facility.

## Case presentation

The child in this case was a 9-year-old female. At 8 years old, she presented with recurrent skin ecchymosis, petechiae, and anemia. A routine blood test revealed pancytopenia. After bone marrow and genetic examinations, she was diagnosed with VSAA and harbored a heterozygous ANKRD26 gene mutation (c.3544A>C/p.S1182R) of paternal origin, with uncertain clinical significance. As the patient was transfusion-dependent, she received a HSCT 3 months after diagnosis, and the HLA matching result was 7/12. Based on our prior experience, we employed a myeloablative conditioning regimen. This regimen consisted of busulfan (0.8mg/kg/day × 2 days, from - 9d to - 8d), cyclophosphamide (60 mg/kg/dose × 2 doses, from - 3d to - 2d), fludarabine (40mg/m^2^/day × 5 days, from - 7d to - 3d), Anti-thymocyte globulin (5 mg/kg/day × 3 days, from - 4d to - 2d). Tacrolimus and mycophenolate mofetil were used for GVHD prophylaxis. The child achieved neutrophil engraftment on day +11 and platelet engraftment on day +17, with 100% donor cells detected in peripheral blood chimerism assessment tests. The hematopoietic reconstruction proceeded smoothly. Subsequently, the patient suffered a pulmonary infection but was discharged after receiving antibacterial treatment.

On day +75, the child was diagnosed with rhinoorbito-cerebral mucormycosis (ROCM), presenting with right ocular swelling, pain, headache, and vision loss. MRI revealed significant right orbital inflammation. Initial empiric therapy (meropenem/vancomycin/voriconazole) was escalated to liposomal amphotericin B (L-AmB) and posaconazole after CSF NGS detected Mucor spp. Due to persistent fever and rising hs-CRP, posaconazole was switched to isavuconazole with L-AmB. Follow-up MRI showed progression to the left orbit and frontal/temporal lobes. On day +145, tacrolimus was discontinued due to severe infection, and bilateral endoscopic debridement confirmed fungal hyphae histopathologically. Infection resolved with negative follow-up NGS (CSF/blood) and resolved MRI lesions, but resulted in permanent bilateral blindness requiring chronic posaconazole suppression.

On day +269 post-HSCT, the patient developed pallor, tachycardia, and lower limb ecchymoses/petechiae after household cold exposure, without other symptoms or GVHD signs. Full donor chimerism was confirmed (day +270). Labs showed macrocytic anemia (Hgb 71g/L, MCV 100fL), marked reticulocytosis (0.086×10^12^/L; normal 0.024-0.084×10^12^/L), and severe thrombocytopenia (nadir 1×10^9^/L). Direct Antiglobulin Test(DAT) was positive, while lactate dehydrogenase (LDH) and indirect bilirubin remained normal. Rhinovirus was detected, while tests for *M. pneumoniae*, fungal markers (G/GM), platelet antibodies, and evidence of Disseminated Intravascular Coagulation (DIC), as well as urine/stool/sputum cultures, were negative. All other tests negative including TA-TMA exclusion criteria: normal ADAMTS13 (Thrombospondin Type 1 Motif, Member 13) activity/antibodies, CFH(Complement Factor H), C5b-9(Complement Component 5b-9), CH50(50% Hemolytic Complement). Bone marrow demonstrated hypercellularity with megakaryocytosis (127 MKs), indicating intact thrombopoiesis. Serum anti-platelet antibodies positive, specific to platelet membrane protein IX(GPIX) and granule membrane protein (GMP140), confirming post-HSCT IMCs.

Initially, thrombocytopenia presented solely with cutaneous hematomas, petechiae, and mild oral mucosal bleeding. Progressively, recurrent epistaxis developed, requiring packed red blood cell (PRBC) transfusions. Despite repeated platelet transfusions, the child exhibited platelet transfusion refractoriness (PTR). Hemostatic interventions included oral etamsylate, Yunnan Baiyao (a traditional Chinese hemostatic agent), gargling with diluted norepinephrine-thrombin solution, and prothrombin complex concentrate administration. Concurrent antimicrobial therapy involved cefoperazone-sulbactam for bacterial infection and posaconazole for fungal prophylaxis.

**Figure 1:**
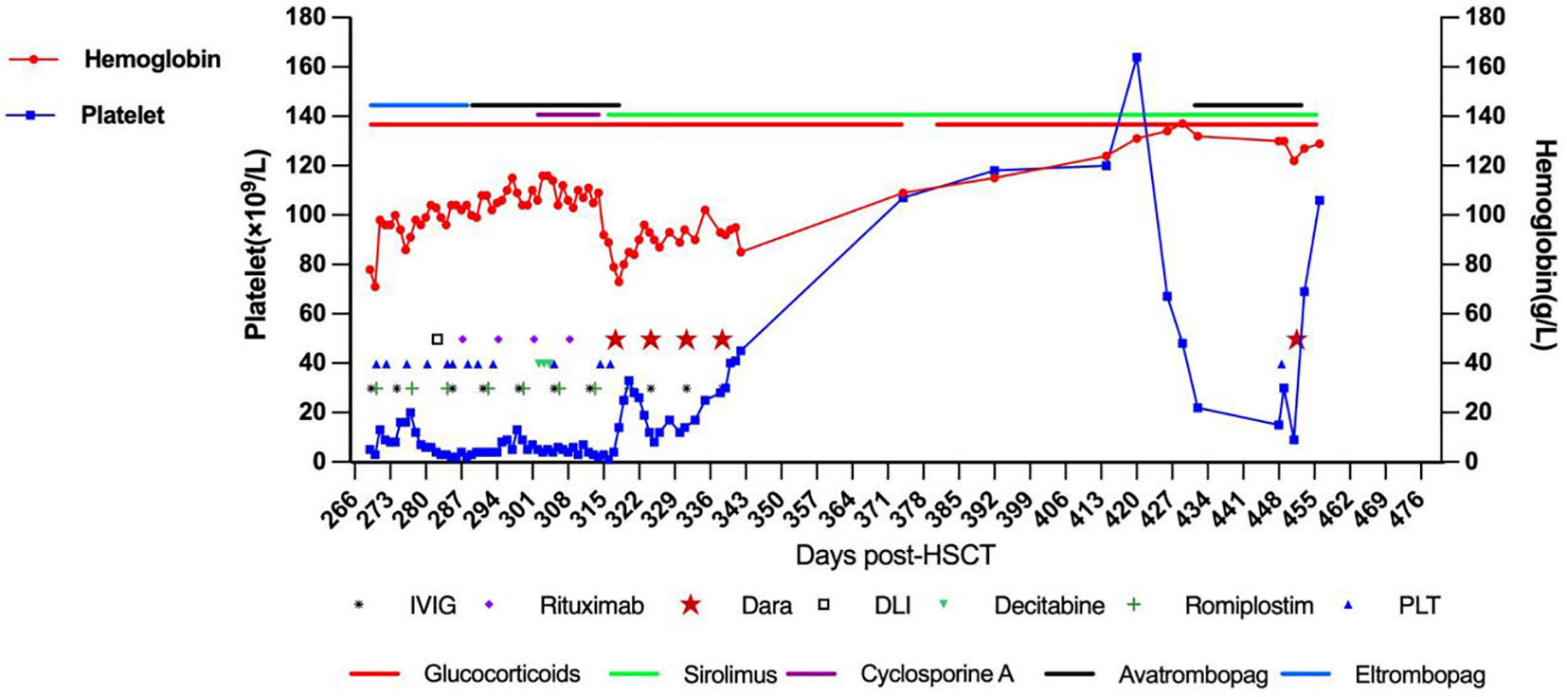
The treatment timeline is presented, demonstrating the patient’s hemoglobin levels, platelet counts, and the various treatments administered over time.

Prednisolone was initiated (2mg/kg/day for 14 days, tapered to 0.4mg/kg/day maintenance) with weekly IVIG (1g/kg ×12 doses). GVHD prophylaxis continued with cyclosporine A (150-200ng/ml) or sirolimus (8-12ng/ml troughs). Platelets remained critically low (2-19×10^9^/L) despite irradiated platelet transfusions. Multiple agents were trialled: eltrombopag (50-75mg/day ×19d) (20), avatrombopag (30mg/day ×1m) (21), romiplostim (10μg/kg/week ×8 doses) (22), oseltamivir (75mg bid ×8d) (23), acetylcysteine (200mg bid ×1m) (24), and decitabine (3mg/m² ×3 doses) (25). Following steroid failure, donor lymphocyte infusion was administered at day +292 (CD3+ cells 1.156x10^6^/Kg, MNC 2.7x10^7^/Kg) with rituximab from day +297 (375 mg/m^2^/w x 4w). Hemoglobin stabilized at 98-116g/L, but platelet counts showed no improvement (**Figure 1**).

**Figure 2:**
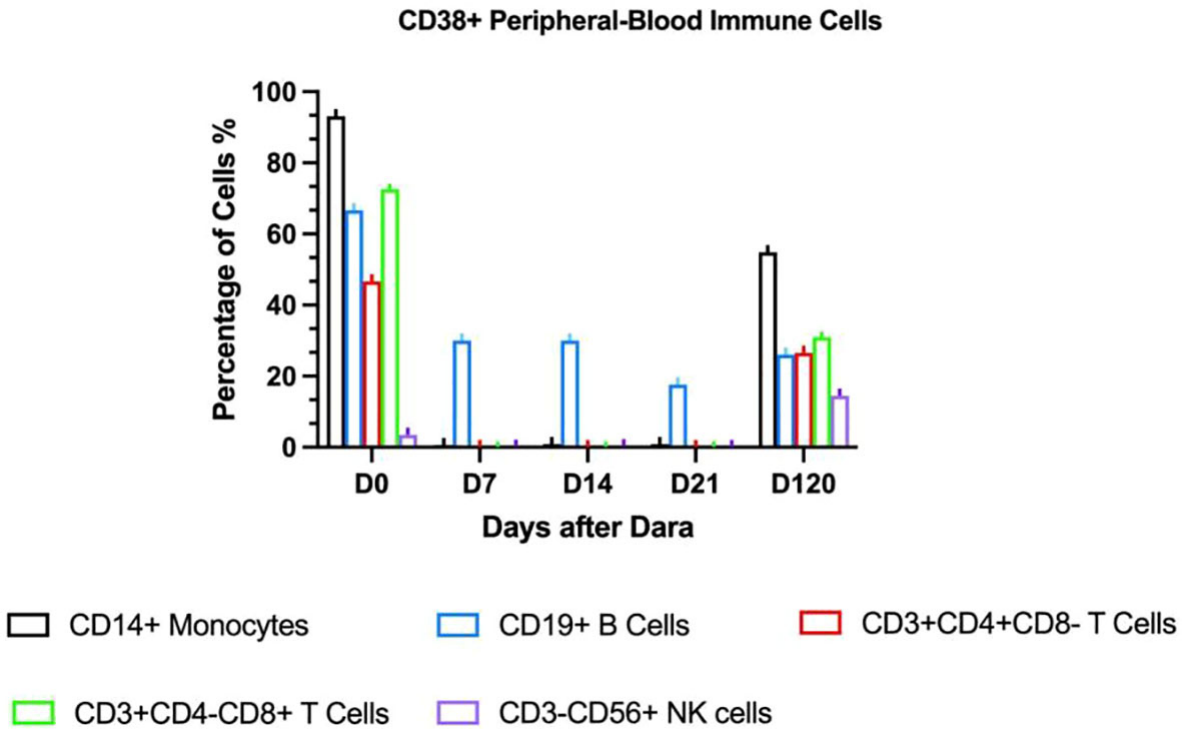
CD38 expression in peripheral blood immune cells.

Given persistent severe thrombocytopenia despite rituximab-induced B-cell depletion, plasma cell-derived anti-platelet antibodies were suspected. Daratumumab (16mg/kg IV weekly) was initiated On day +317 (day +49 after onset of IMT). Platelets rose to 33×10^9^/L post-first dose but declined to a nadir of 8×10^9^/L. After the second dose, counts progressively increased. Four total doses were completed per protocol. Discharge labs showed hemoglobin (Hb) 85g/L and platelets 45×10^9^/L; all infusions were well-tolerated. Pre-treatment peripheral blood revealed elevated CD38 expression on immune cells (T lymphocytes, monocytes, natural killer cells) and increased antibody-secreting cells. Post-CD38-targeting, CD38 expression markedly decreased (**Figure 2**). Platelet (120×10^9^/L) and Hb (120g/L) normalized by day +85. At 3-month follow-up, the child maintained normal hematologic parameters.

**Figure 3:**
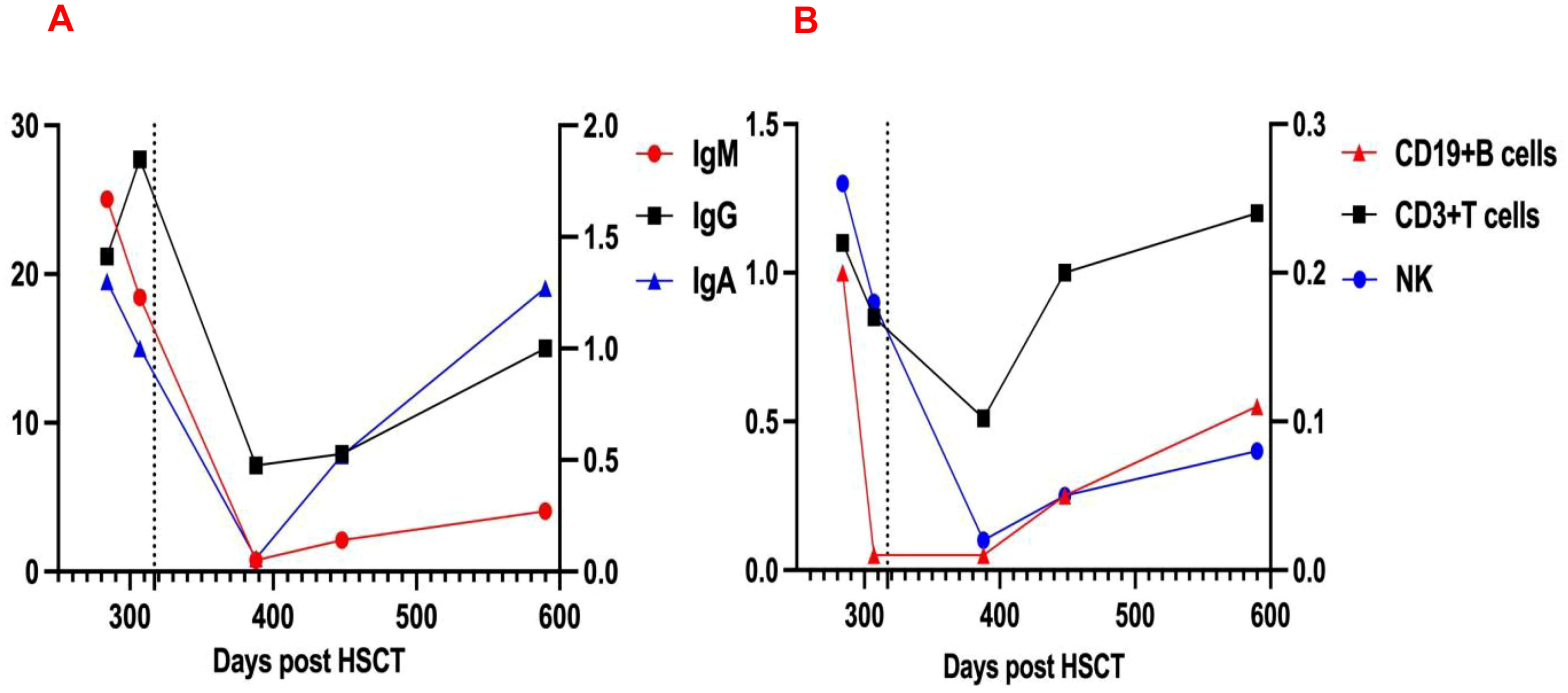
Changes in immunoglobulin **(A)** and lymphocyte counts **(B)** Levels Pre- and Post-Dara, dashed line indicates initiation of daratumumab therapy. IgG and T-lymphocyte counts correspond to the left vertical axis; IgM, IgA, B-lymphocyte, and NK-cell counts are scaled to the right vertical axis.

On day +426, recurrent fever coincided with rhinovirus infection and methylprednisolone tapering. Progressive thrombocytopenia prompted prednisone escalation and avatrombopag initiation. Despite this, platelets fell to 15×10^9^/L with recurrent epistaxis, requiring transfusion; post-transfusion levels only rose marginally to 30×10^9^/L. CD38 expression analysis revealed significant upregulation on immune cells (**Figure 2**), suggesting recurrent plasma cell-derived anti-platelet antibodies. A subsequent daratumumab dose (16mg/kg) on day +451 achieved platelet recovery to 106×10^9^/L within six days. At last follow-up (Dara+341), hemoglobin and platelets normalized without medications, though delayed cellular immunity recovery necessitated IVIG substitution (**Figure 3**). IVIG was administered weekly×12 consecutive weeks, then biweekly×3 months, transitioning to monthly until 12 months post-daratumumab after confirming normalized immunoglobulins.

## Review of literature

To our knowledge, five previous reports have presented data on six patients with post-HSCT IMT (13, 15, 19, 26, 27). A search for additional cases of IMCs was conducted through PubMed, using the keyword “daratumumab” combined with “HSCT, AIC, AIHA, hemolytic, Evans syndrome, ITP, thrombocytopenia, immune dysregulation, and autoimmunity (excluding multiple myeloma)”. Prior to dara initiation, the patient’s hemoglobin had normalized, but refractory immune-mediated thrombocytopenia (IMT) persisted, prompting our review of previous IMT diagnostic cases. The identified articles were screened to exclude duplicate patients and verify cross-references.

To date, including the patient in this study, a total of 7 cases of IMT following HSCT have been reported. Among these, 3 presented with isolated IMT, while 4 were diagnosed with IMT concomitant with autoimmune hemolytic anemia (AIHA). Of the 7 patients, 4 were children (all <12 years of age) with the youngest aged 2 years, and 3 were adults (age 23–60 years, median 25). The primary disease leading to HSCT was myelodysplastic syndrome (n=1), severe aplastic anemia (n=2), primary myelofibrosis (n=1), or a primary immunodeficiency disease (n=3). Two patients received peripheral blood stem cells (PBSC), three whole bone marrow, and in one case the stem cell source was not reported. The child in our case received bone marrow and peripheral blood hematopoietic stem cells. Two patients were transplanted from a 10/10 and two from a 9/10 unrelated donor. One patient was transplanted from a family donor. The child in our case received the transplatation from her father with 7/12 HLA-matched. Chimerism was 100% donor in four and >90% in one patient. The onset of IMT was at a median of day +151 (range +61 to +359), and the first dose of daratumumab was administered at a median of 68 days (range 8–223 days) after IMCs diagnosis. One patient recieved single dose of daratumumab, and daratumumab was administered once weekly at a dose of 16mg/kg in the other cases. The median number of infusions was 4 (range 1-6). Patients had previously zero to nine different therapies, most commonly corticosteroids, rituximab, and IVIG (**Table 1**). In six patients, at least one concomitant treatment was given during daratumumab therapy. Following daratumumab administration, one patient experienced no adverse reactions, while one developed hypertension and chest pain. Two patients exhibited bronchial obstruction, and three developed hypogammaglobulinemia-two of whom concurrently presented with lymphopenia (**Table 2**). No severe adverse events occurred, indicating favorable tolerability. Among these, three patients received regular intravenous immunoglobulin (IVIG) infusions post-daratumumab therapy for infection prophylaxis. Five patients reached transfusion independency and normal blood count. One patient did not respond to daratumumab and proceeded to second HSCT 9 days from first dose of daratumumab. Another one did not respond to daratumumab and deceased from cardiac arrest due to acute heart failure most likely related to pulmonary embolism. The median follow-up time after daratumumab treatment among the six patients who reached transfusion independency was 4 months (range 1–16 months), and six patients were alive without severe long-term toxicities (**Table 2**).

**Table 1:** Clinical summary of seven patients with IMT following HSCT who received daratumumab treatment.

Patient ID (ref.)	Age (years)	Gender	Graft	Donor	ABO mismatch	Chimerism (% donor cells)	Underlying diagnosis	Type of IMCs	Onset of IMT (Days post-HSCT)	Frist Daratumumab dose	
										Days post-HSCT	Days after onset of IMT
1 (12)	23	NA	NA	NA	NA	NA	Chronic granulomatous disease	IMT	NA	NA	8
2 (26)	25	F	BM	MFD (n.a)	+	Mixed	SAA	IMT+AIHA	359	427	68
3 (18)	2	M	PBSC	MUD (9/10)	–	100%	Primary myelofibrosis	IMT+AIHA	AIHA 105; IMT 147	328	223
4 (25)	60	M	PBSC	MUD (10/10)	–	98%	HR-MDS	IMT	166	320	154
5 (14)	10	F	BM	MUD (10/10)	+	100%	LRBA deficiency	IMT	61	118	63
6 (14)	6	M	BM	MUD (9/10)	–	100%	CD40L deficiency	IMT+AIHA	AIHA 58; IMT 83	184	101
7^(this case)^	9	F	BM+PBSC	mMRD(7/12)	–	100%	VSAA	IMT+AIHA	AIHA/IMT 269	317	49

IMT, immune-mediated thrombocytopenia; AIHA, Allo- or autoimmune hemolytic anaemia; HLA, Human leukocyte antigen; HR-MDS, High risk myelodysplastic syndrome; HSCT, Hematopoietic stem cell transplantation; IVIG, i.v. immunoglobulin G; LRBA, Lipopolysaccharide-responsive and beige-like anchor protein; NA, Not available; PBSC, Peripheral blood stem cells; SAA, Severe aplastic anemia; UD, Unrelated donor. Donor type: matched family donor (MFD) or matched unrelated donor (MUD) or mismatched related donors (mMRD). HLA match was indicated where available; e.g. 10/10. If not further specified by the authors marked with n.a.

**Table 2:** Details of daratumumab schedule, durability of response and patients’ status at the last follow-up.

Patient ID (ref.)	Prior treatment	Number of daratumumab doses at 16 mg/kg i.v	Schedule of daratumumab dosing	Adverse effects of daratumumab during/after infusion	Number of daratumumab administrations prior to response	Follow-up time		Patient status at the last follow-up			Remark
						Time post-HSCT	Time post daratumumab administration	Alive/Death	Relapse of IMT after achieving a response	IVIG substitution	
1 (12)	None	1	Single dose	Hypertension and chest pain	None	NA	103 days	Alive	NA	NA	Proceeded to second HSCT 9 days after the first dose of daratumumab
2 (26)	Rituximab/IVIG/Corticosteroids/CSA/Birtezomib	4	Weekly	None	1	508 days	4 months	Alive	No	NA	
3 (18)	Rituximab/IVIG/Corticosteroids/Plasmapheresis/CSA/Abatacept/Bortezomib/Sirolimus	6	Weekly	Hypogammaglobulinemia	1	500 days	6 months	Alive	No	NA	
4 (25)	Vincristine/IVIG/Rituximab/Plasma exchange/Splenectomy/TPO-RAs/Danazol	4	Weekly	Mild lymphopenia, hypogammaglobulinemia	4	28 months	16 months	Alive	No	NA	
5 (14)	Rituximab/IVIG/Corticosteroids/Sirolimus/TPO-RAs/CSA/Tacrolimus	6	Weekly	Nasal congestion and bronchial obstruction	4	14 months	10 months	Alive	No	Yes	
6 (14)	Rituximab/IVIG/Corticosteroids/Plasmapheresis/TPO-RAs/CSA/Eculizumab	3	Weekly	Transient and uncomplicated bronchial obstruction and vomiting	None	Until death	Until death	Death	–	Yes	Deceased from cardiac arrest due to acute heart failure most likely related to pulmonary embolism.
7^(this case)^	Rituximab/IVIG/Corticosteroids/TPO-RAs/CSA/Sirolimus/Oseltamivir/Acetylcysteine/Decitabine/DLI	5	Weekly	Lymphopenia, hypogammaglobulinemia	1	667 days	341 days	Alive	No	Yes	

IVIG i.v., immunoglobulin G; TPO-RAs, thrombopoietin receptor agonists; CSA, Cyclosporin A; DLI, donor lymphocyte infusion; NA, not applicable.

## Discussion

HSCT-associated IMCs pose a substantial threat to patient health and are often extremely challenging to manage. In non-malignant disease settings, several risk factors for developing IMCs post-HSCT have been identified (7). Moreover, environmental factors may play a role. In murine models, inflammatory states have been shown to trigger and promote autoreactive T-cell populations (28), and infections may be linked to lymphodepletion and homeostatic expansion that favor autoreactive clones. The standard first-line treatment for post-HSCT IMCs usually involves corticosteroids and IVIG. For cases unresponsive to steroids and IVIG, rituximab is commonly used as second-line therapy (29). Other treatment approaches reported in the literature include calcineurin inhibitors, sirolimus, mycophenolate mofetil, abatacept, and bortezomib (8). Additional therapeutic strategies include plasmapheresis; chemotherapeutic agents such as 6-mercaptopurine, azathioprine, cyclophosphamide, and vincristine; and in some refractory cases, splenectomy or a second stem cell transplant may be considered (30).

Immune thrombocytopenia (ITP) can be particularly challenging after allogeneic HSCT due to multifactorial causes of thrombocytopenia: GVHD, disease relapse, viral infections, thrombotic microangiopathy, or drug reactions (31). Approximately 40-60% and 20-40% of ITP patients harbor platelet-bound autoantibodies targeting platelet glycoprotein (GP) IIb/IIIa (integrin αIIbβ3) and the GPIb/IX/V complex, respectively (32), with meta-analyses confirming high GP-specific antibody specificity in ITP (33). These antibodies drive platelet destruction via phagocytosis and complement activation (34). To confirm the immunological mechanism underlying thrombocytopenia, detecting glycoprotein-specific anti-platelet antibodies is very useful. In this case, we identified GP IX-specific antibodies and Granule Membrane Protein 140 (GMP140) in the patient without P-selectin (CD62P), which helped diagnose allo-HSCT-related secondary IMT. Reported response rates for post-HSCT IMT are 30-50% after first-line and second-line therapies (15). Similar to literature, our patient showed hemoglobin improvement after multiple interventions (corticosteroids/IVIG/rituximab/oseltamivir/acetylcysteine/decitabine/TPO-RAs), yet thrombocytopenia persisted.

Crickx et al. found that daratumumab may provide clinical benefit in some patients with severe refractory ITP or warm autoimmune hemolytic anemia (AIHA) (35). Koo et al. used daratumumab to treat three patients with relapsing, refractory AIHA following allo-HSCT. Two of the three patients achieved remission and were able to discontinue steroid therapy concurrently (36), Among six reported post-HSCT IMT cases, five failed first-/second-line and multiple interventions therapies (including plasmapheresis), but four achieved complete hematologic remission after 3–6 daratumumab doses, supporting its efficacy in refractory IMT with or without AIHA. This provides further evidence for the potential of daratumumab in treating post- HSCT IMT,similar to our findings in the present case. One patient showed no response and developed severe AIHA one week after the first daratumumab administration. He received salvage therapy with high-frequency plasmapheresis, methylprednisolone pulses, three doses of IVIG, and one dose of eculizumab. Without any response to this therapy, this patient deceased from cardiac arrest due to acute heart failure most likely related to pulmonary embolism (15). Another patient, upon diagnosis of IMT, bypassed first- and second-line therapies and directly proceeded to a single infusion of daratumumab, but exhibited no therapeutic response. He had previously undergone allogeneic HSCT for chronic granulomatous disease with secondary graft failure. It is possible that the lack of observed response could have been due to a low bone marrow reserve following secondary graft failure (13). However, treatment of IMT with daratumumab warrants caution given the lack of long-term experience, the off-label use, and the fact that daratumumab may not always be the best suitable therapy since IMCs can derive from different forms of immune dysregulation (37), not all of which involve plasma cells. Compared to other IMT patients, our case underwent haploidentical transplantation and was diagnosed with ROCM post-transplant. Immunosuppressants were discontinued prematurely, followed by long-term antifungal therapy. This may have contributed to immune dysregulation, potentially triggering IMT development.

Post-HSCT IMT recurrence arises from CD38+ pathogenic plasma cells in spleen/bone marrow that evade CD20-targeted therapies (38). CD38, a receptor widely expressed on plasmablasts, short-lived, and long-lived plasma cells, is an attractive target for the therapeutic antibody daratumumab in IMT treatment (39). Daratumumab has been approved for multiple myeloma (40) and is effective in refractory ITP that have failed second-line treatments (41). Along with the rapid increase in platelet count, the number of CD38+ immune cells, including T lymphocytes, monocytes, CD56dimCD16+ NK (Natural Killer) cells, decreased rapidly within a week after the first daratumumab treatment. Daratumumab infusion effectively suppressed lymphocyte proliferative function (**Figure 2**), indicating its ability to down-regulate autoantibody production by eliminating plasma cells and maintaining a long-term response. Reducing platelet destruction and restoring platelet count may gradually bring the immune activation state back to equilibrium. We observed a significant decrease in CD56+ NK cells, which are important effector cells in antibody-dependent cellular cytotoxicity. In another study, daratumumab plus lenalidomide and dexamethasone (D-Rd) downregulated CD38 expression in multiple myeloma (MM) patients compared to Rd, especially in NK cells, B cells, basophils, monocytes, and CD4+ T cells, with no significant change in CD8+ T cells. Daratumumab-CD38 complex transfer (trogocytosis) reduces surface CD38 levels (42). Effector and memory CD8+ T cells increased, especially in deep responders, showing an activated phenotype with upregulated GrB and HLA-DR (43). Here, we observed decreased CD4+ and CD8+ T cell proportions after daratumumab treatment, potentially related to the non-neoplastic nature of the disease. We hypothesize that daratumumab may lead to rapid platelet recovery by down- regulating antibody-dependent cellular cytotoxicity. Daratumumab shows promise as a treatment option, especially in patients with secondary IMT following allogeneic stem cell transplantation. Four months after the initial daratumumab administration, the percentage of CD38-positive immune cells in the patient’s peripheral blood increased again, coinciding with another drop in platelet levels. Despite this setback, daratumumab treatment was effective once more, highlighting its continued efficacy. This recurrence pattern is a common challenge in managing other forms of immune thrombocytopenia (44). In this study, flow cytometry revealed elevated CD38+ cells, guiding successful daratumumab use. Pre-treatment and intra-treatment assessment of CD38+ cell proportions may help guide the successful use of daratumumab.

CD38 promotes pro-inflammatory phenotypes in innate immune cells, regulates leukocyte recruitment to infected tissues, modulates macrophage phagocytosis and dendritic cell migration to lymph nodes, thereby impairing T/B cell activity. Combined with direct depletion of CD38-expressing NK cells and other immune cells crucial for pathogen control, these features may explain daratumumab-induced immunosuppression in some patients (45). An integrated safety analysis of data pooled from 5 completed phase III, randomized, controlled studies in comparator-treated patients with MM as a first-line in both transplant-eligible and transplant-ineligible patients, and for relapsed/refractory disease, identified high rates of neutropenia, lymphopenia, and pneumonia as common grade 3/4 adverse events with daratumumab compared with comparators (46). In this case, no serious adverse events were observed, and the therapy was well-tolerated, which is consistent with previous research reports (15, 16, 19, 36, 37). Although generally considered a well-tolerated therapy, daratumumab has a significant risk of infectious complications, and this risk has not been fully evaluated in pediatric populations (47). Therefore, its potential hazards should not be ignored in immunocompromised transplant patients. Due to the typically short follow-up periods, it is difficult to determine long-term outcomes, infection frequencies, and potential chronic toxicities, such as impaired humoral immunity. Therefore, we recommend periodic monitoring of cellular and humoral immunity in post-transplant IMT patients receiving daratumumab, alongside tailored immunoglobulin replacement regimens to prevent severe infections.

In conclusion, we present a case study of a pediatric patient with post-HSCT IMCs who achieved complete recovery after treatment with daratumumab. Notably, the treatment was well-tolerated, with the only significant adverse event being prolonged hypogammaglobulinemia. Our findings suggest that daratumumab could be considered as a potential treatment option for managing refractory post-HSCT IMCs.

## Data Availability

The original contributions presented in the study are included in the article/supplementary material. Further inquiries can be directed to the corresponding author.
